# Supplementary material for: β-Cyclocitral Does Not Contribute to Singlet Oxygen-Signalling in Algae, but May Down-Regulate Chlorophyll Synthesis
Source: Plants (Basel). 2022 Aug 19;11(16):2155. doi: 10.3390/plants11162155 (PMC9415740; doi:10.3390/plants11162155)
Supplement: Supplementary file 1 [file plants-11-02155-s001.zip › plants-1777948-supplementary.pdf]

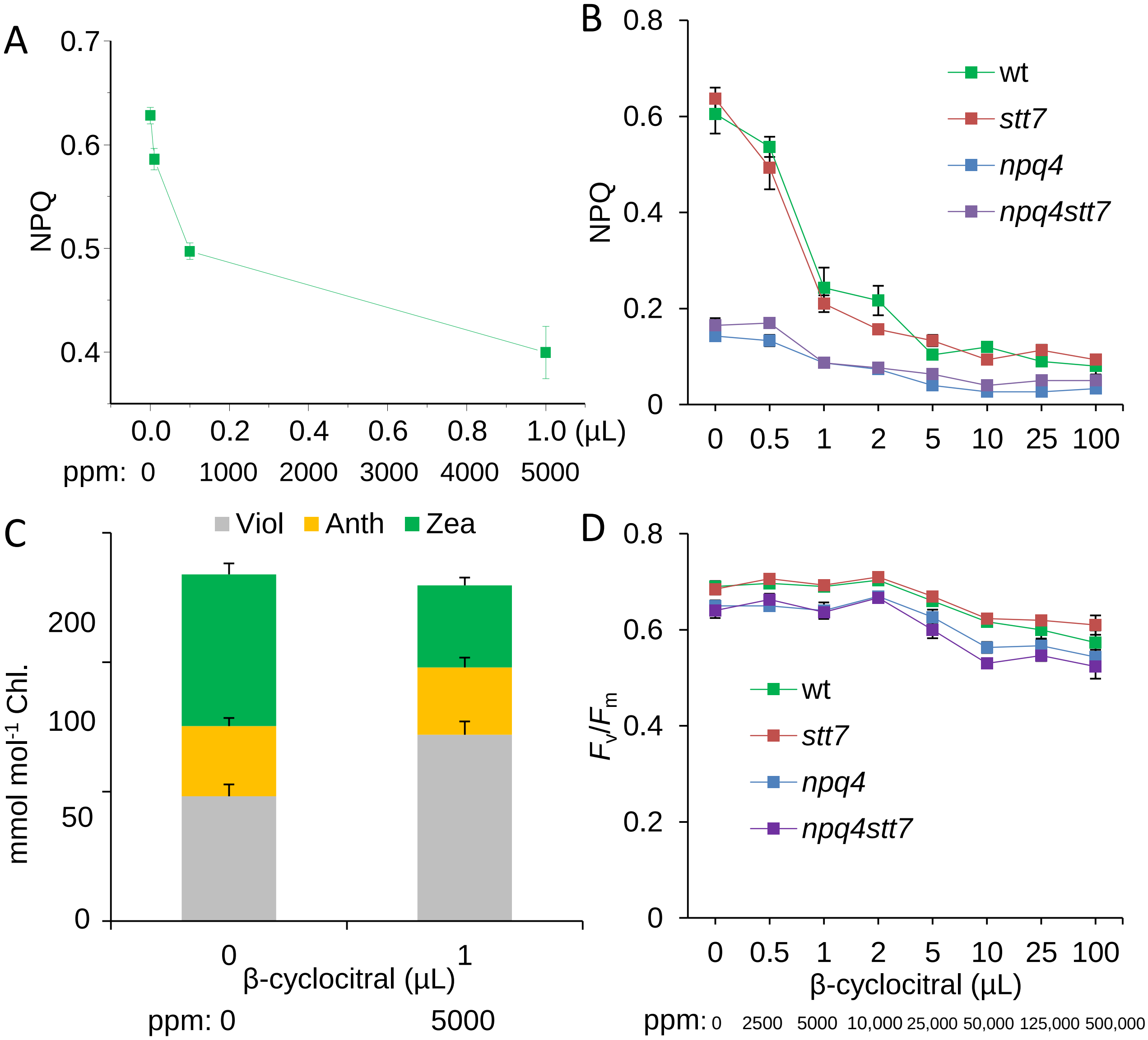

Supplementary Figure S1. Effect of β-cyclocitral treatments on NPQ, the xanthophyll cycle and F_v/F_m in high light-treated cells (A; B) Measurements of NPQ in (A) wild-type 4A (wt) strains, and (B) wt and NPQ mutants, *stt7-9*, *npq4* and *npq4stt7-9* (Allorent et al., 2013), in response to various volumes of β-cyclocitral, as indicated on the X-axis, for 5h at high light (250µmol photons m^{-2} s^{-1}) and 1.5h recovery, n=3±SD. **(C)** Xanthophyll cycle pigments, violaxanthin (Viol), antheraxanthin (Anth) and zeaxanthin (Zea), relative to total chlorophyll amounts (Chl.), in wt cells shown in (A) before 1.5 h recovery. **(D)** F_v/F_m values of the same strains measured in (B). Amounts of β-cyclocitral used for treatment are provided in µL (/Petri dish), and below in corresponding atmospheric parts per million (ppm).

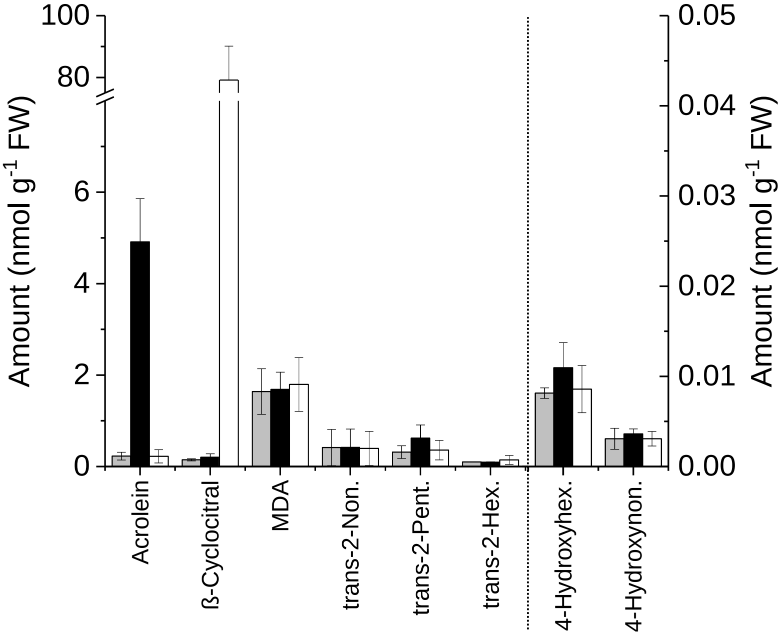

Supplementary Figure S2. Effect of exogenous treatment with acrolein or β-cyclocitral on RES levels. Mixotrophic WT-4A cells grown on 1.5% agar plates at 50 µmol photons m^{-2} s^{-1} were treated with 600 ppm acrolein (black) or β-cyclocitral (white), or untreated (grey). After 2h, 2,4-DNPH-derivatised electrophiles were measured by LC-MS/MS. The right Y-axis scale is for 4-hydroxyhexenal and 4-hydroxynonenal only. All other compounds are on the left Y-axis scale.

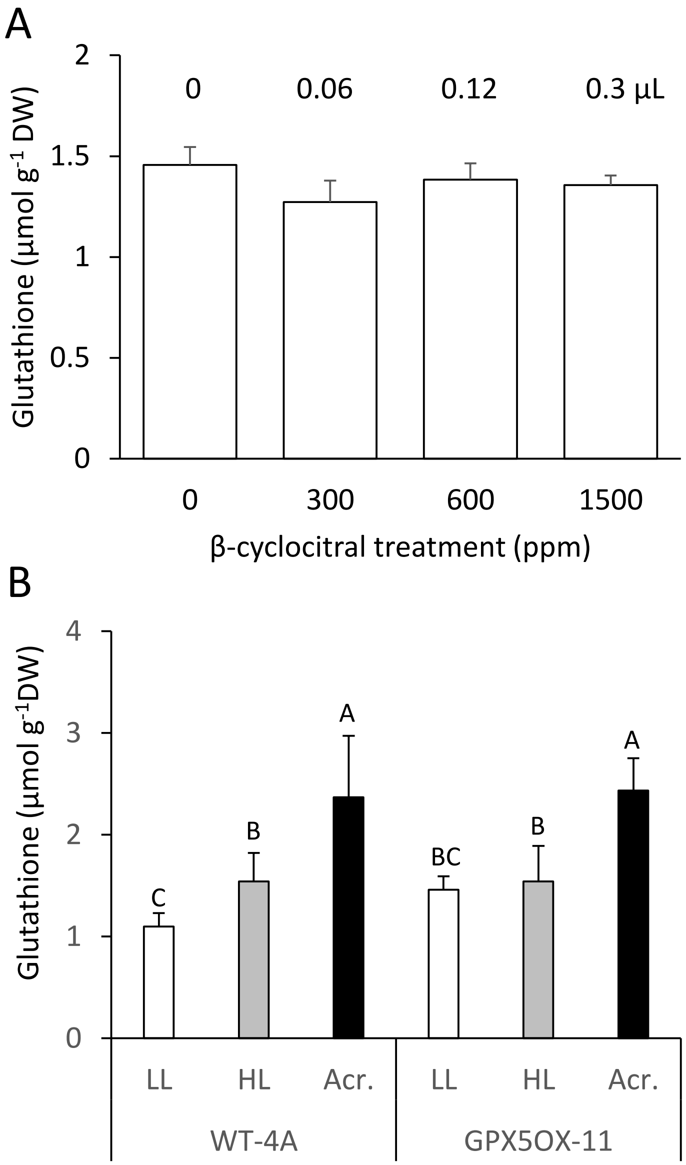

Supplementary Figure S3. Effect of β-cyclocitral, high light (HL) and acrolein treatments on total glutathione (GHS+GSSG) contents. (A) Measurements of WT-4A were made after 4h treatment with the indicated concentration of β-cyclocitral, n= 3 ± SD. The corresponding volume of β-cyclocitral used for treatment is provided above the bars. **(B)** Measurements of WT-4A and *GPXOX-11* after 4h HL or 4h 600 ppm acrolein (Acr.) treatments of low light-acclimated (LL) cells. Unique letters denote significant difference (P<0.05), n= 4 ± SD.

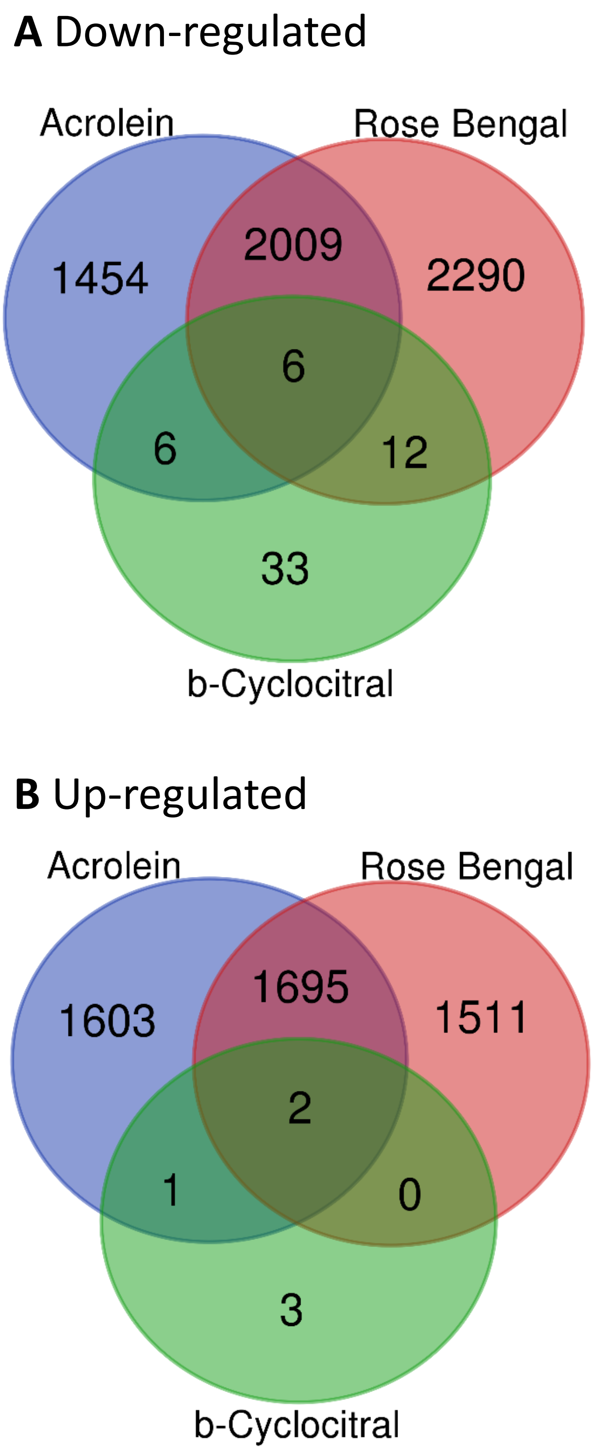

Supplementary Figure S4. Overlap in gene expressional changes between $^1O_2$ stress, acrolein and β-cyclocitral treatments. Overlapping and non-overlapping regions indicate shared and non-shared genes whose transcripts significantly (*P*<0.01, false-discovery rate) decreased **(A)** or increased **(B)** by >2-fold in response to the respective treatment. Venn diagrams were created at http://bioinformatics.psb.ugent.be/webtools/Venn/ and data for Rose Bengal and Acrolein was taken from Roach et al., (2018).

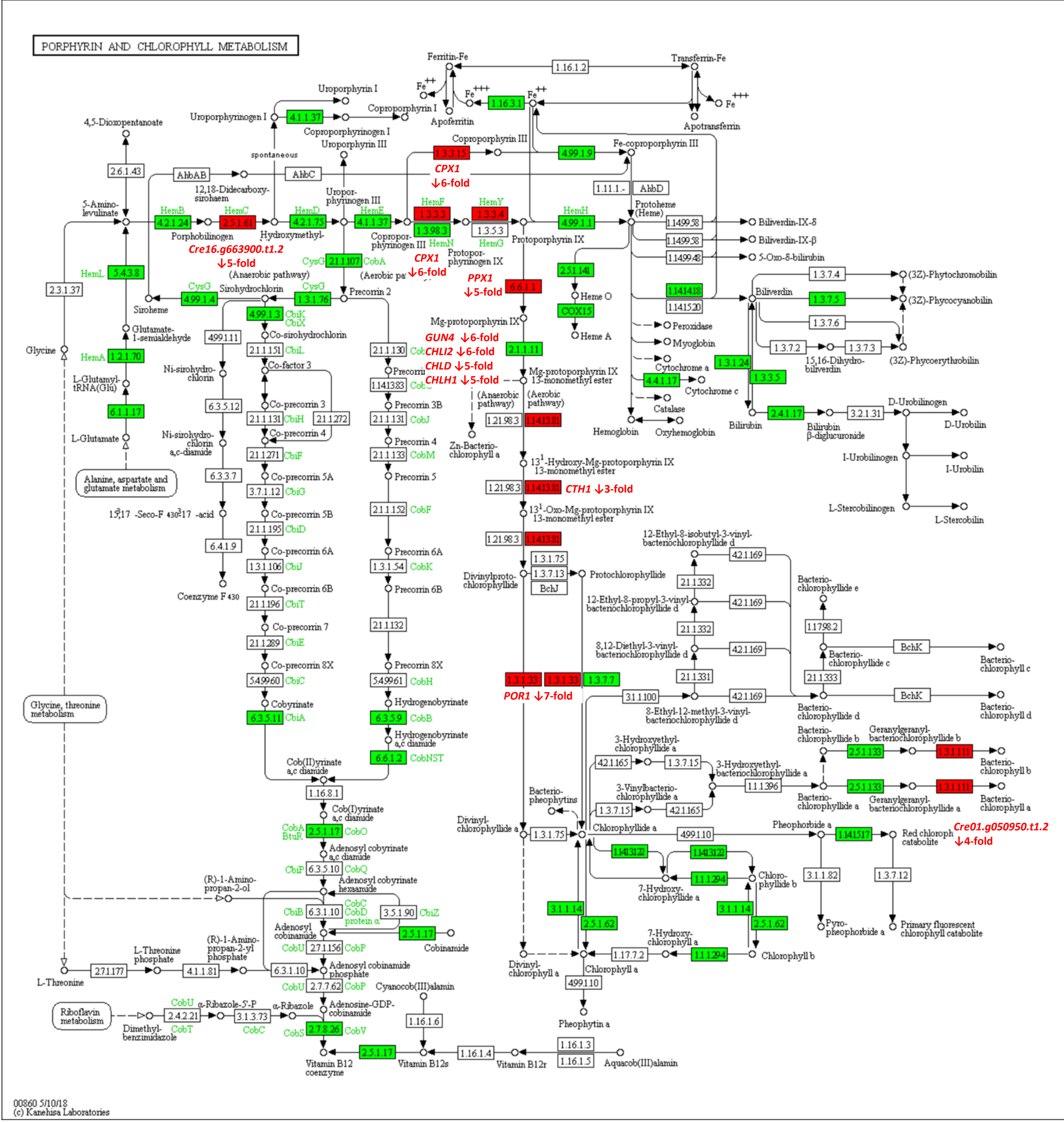

Supplementary Figure S5. Genes with transcription down-regulated by 600 ppm β-cyclocitral that are involved in porphyrin and chlorophyll metabolism. The KEGG scheme shows enzymes involved in porphyrin and chlorophyll metabolism, whereby green boxes shows that *Chlamydomonas reinhardtii* possesses the gene coding for that enzyme that was not differentially expressed, and red boxes denotes that this gene (name in italic red and fold change after arrow) was significantly down-regulated (modified *t*-test *P*<0.01). Data was collected using KEGG https://www.kegg.jp and the Algae Functional Annotation Tool http://pathways.mcdb.ucla.edu/algal/index.html.
